# The Change of System on Its Trial

**Published:** 1915-08-21

**Authors:** 


### THE ARMY CHAPLAIN AND THE R.A.M.C.

### The Change of System on its Trial.

Throughout the Army, as many readers of The Hospital are probably aware, a chaplain of any denomination is invariably addressed and spoken of as "Padre" (pronounced par-dray). As with the A.S.C., the R.A.M.C., and, indeed, all branches and arms of the Service, a large number of temporary commissions have been granted to the Army Chaplains Department; and the regular permanent Army padre is becoming almost a *rara avis* compared with the very large number of those recruited for the duration of the war or for shorter periods.

### Padres with the Medical Units.

In this present campaign (or, rather, in the arrangements previously made in anticipation of any campaign in which the Expeditionary Force might be utilised) it seemed good to those in authority to ordain that all padres should be attached to R.A.M.C. units, instead of to individual combatant battalions and regiments as heretofore. In the original Expeditionary Force—the first six divisions, that is—there was but one padre who escaped this order, according to the gossip of the Force current at the time: this was a highly popular Roman Catholic priest who, though not a Regular Army chaplain, had sufficient influence at the War Office to get permission to be attached to a famous Irish battalion. Since then, it is understood, the rule has been relaxed not infrequently, usually in favour of Roman Catholic padres, for the hierarchy of that Church appears to possess more influence at the War Office than that of any other.

These exceptions notwithstanding, the great majority of the chaplains are still attached to medical units: that is to say, to field ambulances, casualty clearing stations, general hospitals, and so forth.

### A Disputed Point.

This arrangement, now virtually on trial in the British Army, is frequently the subject of discussion, both among the chaplains themselves and among other officers. The writer has heard it debated by infantry and other combatants, as well as by padres and by the messes of the R.A.M.C. Opinions differ considerably as to the relative merits of the old regimental system and the new one; and in point of fact each plan has certain advantages and certain disadvantages of its own. It is evident enough that at a field ambulance or any other medical unit the chaplain has a wide sphere of usefulness: the sick and wounded are brought to his door, as it were, and he can see a far larger number of them than if he were with the infantry in the trenches, where he would be in the way during a fight, and could at best see only a fraction of those needing his ministrations. On the other hand, the chaplain is naturally in closer touch with his parishioners (of the infantry) if he is actually living with them than when his headquarters are with a field ambulance which may be three or four miles away.

The force of this argument in favour of the regimental system is much lessened by the fact that the infantry, though the largest, are by no means the only section of the chaplain's parish: artillery, engineers, R.A.M.C., and all the other troops which constitute a Division, are equally part of his cure, and at a field ambulance he is as a rule better placed for reaching them all than if he were living with the infantry. The ambulances of a Division are seldom far away from the brigades and other formations which compose it: they are usually within easy walking distance, and always within the radius of an hour's ride on horse or bicycle.

### Is the Army Over-Chaplained?

The writer trusts that none of his friends among the padres of many different denominations will take it amiss if he ventures the opinion that the Expeditionary Force in France is at present "over-chaplained." At the outset of the campaign there were usually one, sometimes two, chaplains attached to each field ambulance; and this number, as far as could be judged from a detached standpoint, was amply sufficient for the spiritual needs of the Army at a time when it was much more difficult for a padre to find his scattered and constantly moving flock than it is during the comparatively immobile operations of the last nine months.

Ever since the trench warfare in Flanders was established there has been an incessant stream of chaplains from the base, until at present three or four can be found in every field ambulance. For the most part this plethora of padres is quite unnecessary, as there is seldom enough work to fill their time. Such, at least, is the impression prevalent among most of the R.A.M.C. officers with whom the writer has discussed this topic. There is, of course, plenty of work for one Church of England padre per ambulance (that is, per brigade of infantry with corresponding strength of artillery, engineers, &c.). If there should be an Irish battalion in the brigade, a Roman Catholic padre in addition is advisable; while the inclusion of a Scottish or Ulster battalion will call for the inclusion also of a Presbyterian padre. But it is unusual to find one brigade (of four battalions) containing units from all three countries, whereas it is not uncommon to find in one field ambulance padres of four or five denominations, some of whom have scarcely more than a dozen parishioners.

### What the Padres have done.

So much for the purely administrative side of the Army Chaplains Department. From the personal aspect it is impossible for the writer to do full justice to the work of the padres. Whether it is that he has been exceptionally fortunate or whether service in the Army attracts the very cream of the clergy, or brings out qualities which are sometimes obscured at home, the writer is not prepared to say. But most of the dozen or so of chaplains with whom he has been closely associated have been of the salt of the earth. Church of England, Roman Catholic, Presbyterian, they have been soldiers without forgetting that they are priests, and priests without failing to be sportsmen and gentlemen.

The Clergy in the Ranks.

On the other hand, a certain number of clergy have, to judge by the newspapers, enlisted as orderlies or stretcher-bearers in the R.A.M.C.; the writer has not himself met any of these members of the Churches militant, but he doubts whether they are really doing the best service to their country, though probably the experience of life in the ranks will do them individually nothing but good.

Speaking generally, the most cordial relations exist between the chaplains and the officers of the R.A.M.C. to whose messes they belong. For those chaplains of the Regular Army whom he has met, the writer has the greatest admiration and affection: no more genial, broadminded, and manly set of clerics exist within the British Empire. And of a large proportion of the temporarily commissioned padres very much the same eulogy can be said.

The Relief of Active Service.

Some enlisted padres have rejoiced in their relief from the society of old women of both sexes, to which many of the clergy at home are condemned; to them, and, indeed, to all the padres with the Forces, this terrible war has proved an opportunity, and one they have been quick to avail themselves of and turn to good use. For the manly and the broadminded type of padre there is ample room and a warm welcome at the Front; but the rabid doctrinarians, the fossilised, and the effeminate are not in great demand there, and had much better stay at home.

